# Causal Relationship Between Physical Activity and Thymic Tumors Mediated by Circulating Cytokines: A Mendelian Randomization Mediation Analysis

**DOI:** 10.3390/ijms252413485

**Published:** 2024-12-16

**Authors:** Yulin Sun, Shuaipeng Hao

**Affiliations:** Department of Sports Science, Hanyang University ERICA, Ansan 15588, Republic of Korea; syl0312@hanyang.ac.kr

**Keywords:** physical activity, thymic tumors, circulating cytokines, Mendelian randomization

## Abstract

Physical activity reduces chronic disease risk and enhances immune function, but its causal relationship with thymic tumors—rare neoplasms of the anterior mediastinum—remains unclear. This study investigated whether physical activity reduces thymic tumor risk and whether circulating cytokines mediate this effect. We performed a two-sample Mendelian randomization (MR) analysis using genetic variants as instrumental variables for physical activity and cytokines. Data were obtained from large genome-wide association studies of Europeans, and included the following: physical activity (91,084 individuals), thymic tumors (473,681 individuals with 58 benign and 93 malignant cases), and cytokines (14,824 individuals). The inverse-variance weighted method served as the primary analysis. Genetically predicted physical activity was associated with reduced risks of benign (odds ratio [OR] = 0.381; 95% confidence interval [CI]: 0.158 to 0.921; *p* = 0.032) and malignant thymic tumors (OR = 0.312; 95% CI: 0.155 to 0.628; *p* = 0.001). Mediation analysis identified interleukin-10 receptor subunit β (IL10RB) as a partial mediator, accounting for 5.95% of the protective effect on benign tumors. Sensitivity analyses indicated no pleiotropy or heterogeneity. In conclusion, physical activity causally reduces the risk of thymic tumors, partially mediated by IL10RB, highlighting its potential role in cancer prevention through immunomodulation.

## 1. Introduction

Physical activity is globally recognized for its extensive health benefits, including reducing the risk of chronic diseases and enhancing immune function [1,2,3]. Regular exercise modulates the immune system by influencing the production and circulation of cytokines, i.e., critical mediators of inflammation and immune responses [4,5]. Key cytokines, such as interleukins, interferons, and tumor necrosis factors, play pivotal roles in cell signaling and have been implicated in the pathogenesis of various cancers [6].

Thymic tumors, although relatively rare, are the most common neoplasms of the anterior mediastinum in adults [7,8]. Originating from thymic epithelial cells, these tumors can cause significant morbidity due to their proximity to vital structures and potential for malignant transformation [9]. The etiology of thymic tumors remains poorly understood, and epidemiological data are limited. Emerging evidence suggests that immune dysregulation and chronic inflammation may contribute to their development [10]. We specifically focused on thymic tumors due to the scarcity of established environmental or lifestyle risk factors and the limited epidemiological data. By examining a modifiable factor like physical activity, we aimed to determine whether immune-related pathways could offer opportunities for prevention, even in tumors where such risk factors remain largely unknown.

Despite the known influence of physical activity on immune function and inflammation, the causal relationship between physical activity and thymic tumor risk has not been thoroughly investigated. Moreover, the potential mediating role of circulating cytokines in this relationship is not well defined. Understanding these mechanisms is crucial for developing effective prevention strategies and enhancing our knowledge of tumor immunobiology.

Mendelian randomization (MR) offers a robust methodological approach to assess causal inferences between exposures and outcomes using genetic variants as instrumental variables [11,12]. By leveraging genetic predispositions to higher levels of physical activity and cytokine expression, MR minimizes confounding factors and reverse causation that often plague observational studies [13]. Recent large-scale genome-wide association studies (GWASs) have identified genetic variants associated with habitual physical activity levels, providing genetically informed proxies for long-term differences in activity patterns [14,15]. While environmental and behavioral factors strongly influence physical activity, these genetic variants capture innate predispositions that persist over the life course, allowing MR to infer causality by minimizing confounding and reverse causation. Incorporating mediation analysis within the MR framework allows for the exploration of potential pathways, such as the mediating effect of cytokines, through which physical activity may influence thymic tumor risk [16].

This study aimed to investigate the causal relationship between physical activity and thymic tumors, focusing on the mediating role of circulating cytokines. Utilizing MR mediation analysis, we sought to elucidate the biological mechanisms underlying this relationship. Our findings may provide valuable insights into cancer prevention and contribute to a broader understanding of how lifestyle factors influence tumor development through immune modulation.

## 2. Results

### 2.1. Selected Genetic Instruments

In selecting instrumental variables for physical activity, we identified SNPs significantly associated with physical activity at the genome-wide significance threshold (*p* < 5 × 10^−8^). We assessed each SNP for potential associations with known confounders using the PhenoScanner database, considering factors such as BMI, smoking status, alcohol consumption, and other lifestyle variables that could influence thymic tumor risk.

Three SNPs were excluded due to significant associations with confounding variables (*p* < 5 × 10^−8^). Specifically, rs11012732 and rs59499656 were associated with BMI, and rs34517439 was linked to smoking behavior (details provided in Supplementary Table S1). Removing these SNPs helps satisfy the second core assumption of MR—that the genetic instruments are not associated with confounders of the exposure-outcome relationship.

After exclusion, five independent SNPs were retained as robust genetic instruments for physical activity (listed in Supplementary Table S2). These SNPs were confirmed to be independent (linkage disequilibrium r^2^ < 0.001) and exhibited strong associations with physical activity levels without significant links to potential confounders. The F-statistics for all selected SNPs exceeded 10, indicating sufficient instrument strength and reducing the likelihood of weak instrument bias.

### 2.2. Effects of Physical Activity on Thymic Tumors

The primary inverse-variance weighted MR analysis demonstrated a significant inverse association between physical activity and the risk of thymic tumors. For benign thymic neoplasms, higher physical activity levels were associated with a reduced odds ratio (OR) of 0.381 (95% confidence interval [CI]: 0.158 to 0.921; *p* = 0.032). Similarly, for malignant thymic neoplasms, the OR was 0.312 (95% CI: 0.155 to 0.628; *p* = 0.001). These findings suggest that increased physical activity may significantly lower the risk of both benign and malignant thymic tumors.

Supplementary analyses using MR-Egger regression and the weighted median method supported these results, reinforcing the validity of the observed associations (Table 1). The consistency across different MR techniques enhances the credibility of the causal inference.

Sensitivity analyses were conducted to assess the robustness of the findings and check for potential violations of MR assumptions. Cochran’s Q test revealed no significant heterogeneity among the instrumental variables (*p* > 0.05), indicating that the SNPs used were homogeneous and that the IVW estimates were reliable. The MR-Egger intercept test showed no evidence of directional horizontal pleiotropy (*p* > 0.05), suggesting that the genetic instruments influenced thymic tumor risk primarily through physical activity rather than alternative pathways.

The MR-PRESSO global test did not identify any significant outliers among the included SNPs (Supplementary Table S3), indicating that pleiotropy is unlikely to bias the results. Furthermore, the leave-one-out analysis demonstrated that no single SNP disproportionately influenced the overall effect estimates (Supplementary Figures S1–S3). This analysis involved sequentially removing each SNP and re-estimating the causal effect, confirming the stability and robustness of the associations.

### 2.3. Mediation by Circulating Cytokines

To explore the potential mediating role of circulating cytokines in the relationship between physical activity and thymic tumors, we conducted a two-step MR mediation analysis.

In the first step, we identified which circulating cytokines are causally influenced by physical activity. We performed MR analyses using genetic instruments for physical activity and summary statistics from GWAS on circulating cytokine levels (Supplementary Table S4). Before adjusting for multiple comparisons using the false discovery rate (FDR), our results indicated that physical activity significantly affected the levels of several cytokines, generally decreasing their concentrations. Specifically, higher levels of physical activity were associated with reduced levels of C-C motif chemokine ligand 19 (CCL19), natural killer cell receptor 2B4, T-cell surface glycoproteins CD5 and CD6, fractalkine, interleukin-10 receptor subunit beta (IL10RB), interleukin-15 receptor subunit alpha (IL15RA), interleukin-18 receptor 1 (IL18R1), macrophage inflammatory protein 1-alpha (MIP-1α), matrix metalloproteinase-10 (MMP-10), programmed cell death 1 ligand 1 (PD-L1), and tumor necrosis factor receptor superfamily member 9 (TNFRSF9). Conversely, physical activity was associated with increased levels of cystatin D (Table 2).

After applying FDR correction to control for multiple testing, significant associations persisted between physical activity and reduced levels of IL10RB (β = −0.066; 95% CI: −0.104 to −0.027; *p* = 0.037) and CCL19 (β = −0.065; 95% CI: −0.105 to −0.026; *p* = 0.038). Sensitivity analyses, including MR-Egger regression and the weighted median method, confirmed the robustness of these associations (Supplementary Table S3 and Figures S4–S6).

In the second step, we investigated whether the cytokines influenced by physical activity causally affect thymic tumor risk. Due to the limited availability of SNPs meeting the conventional genome-wide significance threshold (*p* < 5 × 10^−8^), we adopted a more relaxed threshold of *p* < 5 × 10^−7^ to identify SNPs associated with IL10RB and CCL19. The characteristics of these SNPs are detailed in Supplementary Table S5, and the calculated F-statistics indicated sufficient instrument strength, minimizing concerns about weak instrument bias.

The primary IVW MR analysis revealed that higher levels of IL10RB were significantly associated with a reduced risk of benign thymic neoplasms (OR = 2.394; 95% CI: 1.203 to 4.766; *p* = 0.013). Supplementary analyses using MR-Egger regression and the weighted median method supported the consistent direction and magnitude of this effect. However, no significant associations were observed between IL10RB or CCL19 levels and malignant thymic neoplasms (Supplementary Table S6). Sensitivity analyses, including tests for heterogeneity and pleiotropy, confirmed the stability and validity of these findings (Supplementary Table S3 and Figures S7–S9).

Finally, we estimated the indirect effect of physical activity on benign thymic neoplasms mediated through IL10RB levels. By combining the causal estimates from the two MR steps, we calculated that approximately 5.95% of the protective effect of physical activity on benign thymic neoplasms is mediated via reductions in IL10RB levels.

## 3. Discussion

This study provides novel genetic evidence supporting a causal relationship between increased physical activity and a reduced risk of both benign and malignant thymic tumors. Utilizing MR analysis, we demonstrated that higher levels of physical activity are significantly associated with lower odds of developing thymic tumors. Furthermore, our two-step MR mediation analysis indicated that IL10RB may partially mediate this protective effect, suggesting a potential immunomodulatory pathway through which physical activity influences thymic tumor risk.

The primary MR analysis revealed a significant inverse association between physical activity and thymic tumors. Specifically, individuals genetically predisposed to higher physical activity levels had a lower risk of both benign and malignant thymic neoplasms. This finding aligns with extensive epidemiological evidence that regular physical activity reduces the risk of various cancers, possibly through mechanisms such as enhanced immune surveillance, reduced inflammation, hormonal modulation, and improved metabolic profiles [17,18,19].

Our mediation analysis identified IL10RB as a partial mediator in the relationship between physical activity and benign thymic neoplasms. IL10RB is a component of the receptor complex for interleukin-10 (IL-10), a cytokine with potent anti-inflammatory properties [20,21]. IL-10 signaling plays a critical role in regulating immune responses by limiting inflammatory processes and maintaining immune homeostasis [22]. The observed reduction in IL10RB levels associated with increased physical activity suggests that physical activity may modulate IL-10 signaling pathways, potentially enhancing anti-tumor immune responses.

The partial mediation by IL10RB indicates that while physical activity exerts a direct protective effect against thymic tumors, a portion of this effect is mediated through alterations in cytokine signaling. Approximately 5.95% of the protective effect on benign thymic neoplasms was mediated via reductions in IL10RB levels. This suggests that other mechanisms, such as improved immune function, hormonal changes, and metabolic adaptations, also contribute significantly to the reduced cancer risk associated with physical activity.

Furthermore, our study did not include a control population with known genetic predispositions (e.g., MEN1 syndrome) to thymic tumors. Examining the IL10RB status and physical activity patterns in such a genetically driven subgroup could provide more direct evidence of IL-10-related protective mechanisms. Future research using MEN1 patient cohorts or other genetically predisposed populations may offer deeper insights into the specificity and generalizability of our findings.

While the protective effects of physical activity on cancer risk are well documented, specific studies on thymic tumors are limited due to their rarity [23]. Our findings extend the current understanding by highlighting a potential causal relationship between physical activity and reduced thymic tumor risk. Previous research has shown that physical activity can modulate cytokine profiles, leading to decreased levels of pro-inflammatory cytokines and alterations in anti-inflammatory cytokines like IL-10 [24,25].

The role of IL-10 in cancer is complex, as it can exhibit both anti-tumor and pro-tumor effects depending on the context [26]. IL-10 can suppress anti-tumor immune responses by inhibiting the activation of macrophages and dendritic cells, leading to reduced antigen presentation and T-cell activation [27]. Therefore, decreased IL10RB expression may enhance immune surveillance by diminishing IL-10-mediated immunosuppression in the tumor microenvironment. Mechanistically, IL10RB, together with IL10RA, forms the IL-10 receptor complex, which, upon IL-10 binding, activates the JAK-STAT signaling pathway—particularly STAT3—to induce anti-inflammatory and immunosuppressive gene expression. By reducing IL10RB levels, physical activity may limit IL-10-driven immunosuppressive signaling, thereby shifting the tumor microenvironment towards a state more conducive to effective anti-tumor immunity. Our findings suggest that physical activity may modulate IL-10 signaling, thereby promoting a more effective anti-tumor immune response.

A key strength of this study is the use of MR analysis, which leverages genetic variants as instrumental variables to infer causality, minimizing the confounding and reverse causation inherent in observational studies [28]. The two-sample MR design allowed us to utilize large, independent GWAS datasets, increasing the robustness and generalizability of our findings [29].

However, several limitations should be acknowledged. First, the rarity of thymic tumors resulted in a relatively small number of cases (58 benign and 93 malignant), which may affect the statistical power and precision of our estimates. Second, our analysis was confined to individuals of European ancestry, potentially limiting the applicability of the results to other ethnic groups. Third, while we conducted extensive sensitivity analyses to test the MR assumptions, the possibility of residual pleiotropy cannot be entirely excluded. Although the MR-Egger intercepts and MR-PRESSO global tests did not indicate significant pleiotropy, undetected horizontal pleiotropy may bias the results [30].

In the mediation analysis, the limited availability of strong genetic instruments for IL10RB required a more lenient genome-wide significance threshold (*p* < 5 × 10^−7^), which may introduce weak instrument bias [31]. Despite calculating F-statistics to ensure instrument strength, the results should be interpreted with caution. Additionally, the proportion of the effect mediated by IL10RB was relatively small, indicating that other mediators and pathways likely contribute to the protective effect of physical activity.

Moreover, while Mendelian randomization provides a valuable tool for causal inference, it does not fully replicate a controlled epidemiological setting with well-defined reference groups. The absence of specific control populations, such as those with distinct environmental exposures or genetically predisposed cohorts, limits our ability to directly compare outcomes across clearly defined subpopulations. Thus, our results must be interpreted with caution, recognizing that MR leverages genetic proxies rather than direct exposure measurements.

Despite these limitations, our study’s strength lies in its robust methodological design and the use of large-scale genetic datasets. By employing MR, we reduced the influence of confounding and reverse causation, providing more reliable causal estimates. Moreover, our findings highlight the potential biological mechanisms—namely IL10RB modulation—involved in the protective effect of physical activity.

From a real-world perspective, understanding the causal pathways linking physical activity, cytokine signaling, and thymic tumor risk can inform targeted prevention strategies and public health recommendations. Encouraging regular exercise could complement other cancer prevention measures, and insights into IL-10 signaling pathways may guide the development of exercise-based interventions or pharmacological agents that emulate the immune-modulating benefits of physical activity. Ultimately, these findings could contribute to more personalized and effective approaches to cancer prevention and management.

In addition, beyond prevention, physical activity may improve patient outcomes, enhance treatment tolerance, and potentially influence therapeutic responses in individuals who develop cancer. These broader benefits underscore the multifaceted role of physical activity in cancer management and survivorship.

Our findings have important implications for public health and cancer prevention strategies. They suggest that promoting physical activity may reduce the risk of thymic tumors, adding to the list of cancers for which physical activity is protective. Given the challenges in diagnosing and treating thymic tumors due to their location and potential for malignancy, preventive measures are particularly valuable.

The identification of IL10RB as a partial mediator highlights the role of immune modulation in the anti-cancer effects of physical activity. This insight opens avenues for future research to explore IL-10 signaling pathways as potential targets for therapeutic interventions. Understanding how physical activity influences cytokine networks could lead to the development of exercise-based interventions or pharmacological agents that mimic these effects.

Future studies should aim to replicate our findings in larger and more diverse populations to enhance generalizability. Longitudinal studies and randomized controlled trials investigating the impact of different types, intensities, and durations of physical activity on thymic tumor risk and cytokine profiles would provide more detailed insights. Additionally, exploring interactions between genetic predisposition, environmental factors, and lifestyle behaviors could inform personalized cancer prevention strategies.

## 4. Materials and Methods

### 4.1. Study Design

**Figure 1:**
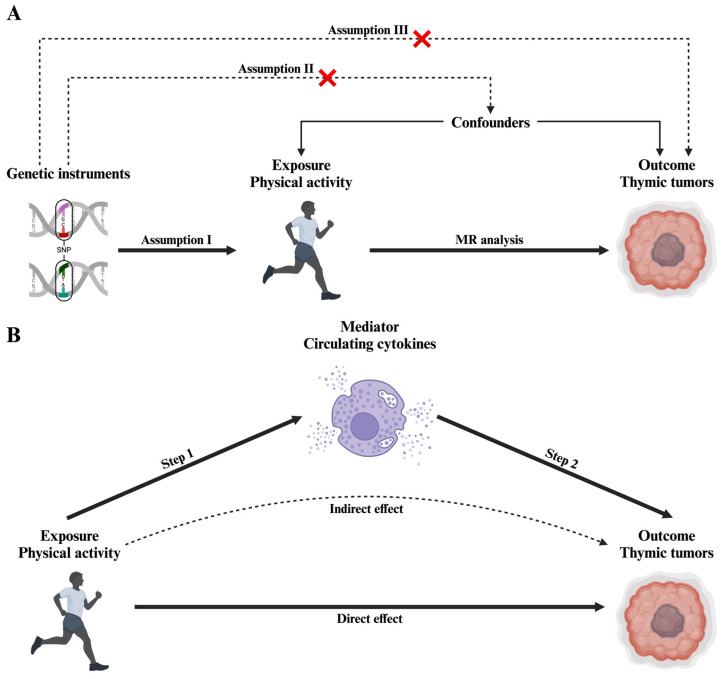
Schematic diagram of the study design. (**A**) MR analysis satisfying three key assumptions. (**B**) Two-step MR analysis framework.

We conducted a two-sample MR analysis to investigate the causal effect of physical activity on thymic tumors and a two-step MR to assess the mediating role of circulating cytokines (Figure 1). The MR analysis relies on three key assumptions: (i) the genetic instruments are robustly associated with exposure (physical activity); (ii) the instruments are not associated with confounders; and (iii) the instruments affect the outcome (thymic tumors) only through exposure [32,33,34].

In the first step, we assessed the direct causal effect of physical activity on thymic tumors using genetic variants as instrumental variables (Figure 1A). In the second step, we evaluated the effect of physical activity on circulating cytokine levels and then the effect of these cytokines on thymic tumor risk (Figure 1B).

### 4.2. Data Sources

We obtained summary statistics for physical activity from a GWAS within the UK Biobank cohort, involving 91,084 participants of European ancestry [14]. The UK Biobank is a large-scale biomedical database containing genetic and health information from over 500,000 individuals aged 40–69 years. Physical activity levels were objectively measured using wrist-worn triaxial accelerometers (Axivity AX3) over a continuous 7-day period. Raw acceleration data at 100 Hz were processed to remove gravity and sensor noise, calibrate device sensitivity, and identify wear periods, resulting in reliable estimates of physical activity metrics.

Summary statistics for thymic tumors were derived from the FinnGen consortium (Release 11), which integrates genotype data from Finnish biobanks with comprehensive health registry data [35]. The dataset includes 473,681 individuals and over 21 million genetic variants. We extracted data for benign neoplasms of the thymus (phenocode: CD2_BENIGN_THYMUS; 58 cases and 453,675 controls) and malignant neoplasms of the thymus (phenocode: C3_THYMUS_EXALLC; 93 cases and 345,118 controls). Genotyping was performed using customized arrays, and imputation utilized a population-specific reference panel (Finland-legacy). Analyses were adjusted for age, sex, genotyping batch, and genetic population structure to minimize confounding.

Circulating cytokine data were sourced from a large-scale proteome-wide association study that identified genetic variants influencing the levels of 91 plasma cytokines [36]. The study aggregated data from 11 cohorts, totaling 14,824 participants of European descent. Cytokine concentrations were measured using the Olink Target 96 multiplex panels, ensuring high specificity and sensitivity. Quality control procedures included normalization across batches and cohorts. Genome-wide association analyses were performed to identify protein quantitative trait loci (pQTLs) associated with cytokine levels. Summary statistics are publicly available in the GWAS Catalog under identifiers GCST90274758 to GCST90274848.

All datasets comprised individuals of European ancestry to minimize population stratification bias. Ethical approval and informed consent were obtained by the original study investigators. This research utilized publicly available summary statistics; no additional ethical approvals were required for secondary data analysis.

### 4.3. Selection of Genetic Instruments

To identify single-nucleotide polymorphisms (SNPs) significantly associated with physical activity and circulating cytokine levels, we adhered to rigorous selection criteria based on standard practices in MR studies. For both the exposure (physical activity) and the mediators (circulating cytokines), we selected SNPs that reached the genome-wide significance threshold of *p* < 5 × 10^−8^ in their respective GWAS [37]. This stringent threshold minimizes the likelihood of false-positive associations, ensuring the reliability of our instrumental variables.

To ensure independence among the selected SNPs and reduce linkage disequilibrium (LD), we performed LD clumping using PLINK v1.9 software with an r^2^ threshold of 0.001 and a clumping window size of 10,000 kilobases (kb) [38]. This process retains the most strongly associated SNPs while eliminating those in high LD, preventing redundancy and potential biases in the MR analysis.

We carefully examined palindromic SNPs with ambiguous allele orientations (e.g., A/T or G/C alleles). SNPs with minor allele frequencies close to 50% were excluded to avoid strand alignment ambiguities across different datasets. This precaution helps maintain accurate allele matching between the exposure and outcome GWAS, which is critical for valid MR estimates [39].

To mitigate potential confounding due to pleiotropy, we screened the selected SNPs against the PhenoScanner database and relevant GWAS catalogs for associations with potential confounders [40], such as body mass index (BMI), smoking status, alcohol consumption, and other lifestyle factors, which have been broadly linked to cancer risk, though their specific associations with thymic tumors remain unclear [41,42,43]. The SNPs significantly associated with these confounders (*p* < 5 × 10^−8^) were excluded from our instrument sets to enhance their specificity to the exposure of interest.

We assessed the strength of our instrumental variables by calculating the F-statistic for each SNP using the following formula: F = R^2^ (N − K − 1) / K (1 − R^2^), where R^2^ is the proportion of variance in the exposure explained by the SNP, N is the sample size, and K is the number of instruments. An F-statistic greater than 10 was considered indicative of a strong instrument, reducing the risk of weak instrument bias in our causal estimates [44].

### 4.4. Statistical Analysis

The primary analytical method in this MR study was the inverse-variance weighted (IVW) approach, which provides a consistent estimate of the causal effect when all genetic instruments satisfy MR assumptions [37]. To enhance the robustness of our causal inference, we also applied supplementary MR methods, including MR-Egger regression and the weighted median estimator [33,45]. These methods offer different sensitivities to violations of MR assumptions, particularly regarding horizontal pleiotropy. A causal effect was considered statistically significant if the IVW-derived *p*-value was less than 0.05 and the effect estimates from all MR methods were consistent in direction [46].

To assess the validity of our findings and detect potential pleiotropy or heterogeneity among the instrumental variables, we conducted several sensitivity analyses. Heterogeneity across SNPs was evaluated using Cochran’s Q statistic within the IVW framework [47]. Horizontal pleiotropy was assessed using the intercept from the MR-Egger regression, where a nonzero intercept indicates potential pleiotropic effects [33]. Additionally, we employed the MR Pleiotropy RESidual Sum and Outlier (MR-PRESSO) test to identify and correct for outlier SNPs that might bias the causal estimates due to pleiotropy [30]. A leave-one-out analysis was performed, systematically excluding each SNP to examine its influence on the overall causal estimate and ensure that the results were not driven by any single instrumental variable [31].

For the mediation analysis, we utilized a two-step MR approach to estimate the indirect effect of physical activity on thymic tumors mediated through circulating cytokines. In the first step, we estimated the causal effect of physical activity on each cytokine (β_1_). In the second step, we estimated the causal effect of cytokines influenced by physical activity on thymic tumor risk (β_2_). The indirect effect was calculated as the product of these two estimates (β_indirect = β_1_ × β_2_). The proportion mediated was determined by dividing the indirect effect by the total effect of physical activity on thymic tumors (β_total), obtained from the initial MR analysis (proportion mediated = β_indirect/β_total). Confidence intervals for the indirect effects and proportion mediated were calculated using the delta method, which accounts for the uncertainty in both β_1_ and β_2_ estimates [48,49].

To control for multiple testing and reduce the likelihood of false-positive results, we applied the FDR correction using the Benjamini-Hochberg procedure [50]. Statistical significance was established at an adjusted *p*-value of less than 0.05.

All statistical analyses were conducted using the TwoSampleMR (version 0.6.5) and MRPRESSO (version 1.0) packages in R (version 4.2.3).

## 5. Conclusions

In conclusion, this study provides genetic evidence supporting the causal protective effect of physical activity on thymic tumors, with IL10RB partially mediating this relationship. These findings enhance our understanding of the biological mechanisms through which physical activity influences cancer risk, emphasizing the importance of regular exercise in cancer prevention strategies. Encouraging physical activity as a modifiable lifestyle factor may offer a viable approach to reduce the incidence of thymic tumors. Further research is warranted to confirm these results and explore the potential for targeted interventions that leverage immune modulation to prevent or treat thymic tumors.

## Figures and Tables

**Table 1:** MR analysis results of physical activity on thymic tumors.

Outcome	Method	OR (95% CI)	SE	*p*-Value
Benign thymic neoplasms	Inverse-variance weighted	0.381 (0.158, 0.921)	0.450	0.032
	Weighted median	0.445 (0.145, 1.363)	0.571	0.156
	MR-Egger regression	0.059 (0.002, 1.484)	1.647	0.184
Malignant thymic neoplasms	Inverse-variance weighted	0.312 (0.155, 0.628)	0.357	0.001
	Weighted median	0.258 (0.104, 0.639)	0.463	0.003
	MR-Egger regression	0.540 (0.041, 7.090)	1.314	0.671

**Table 2:** Main results of MR analysis for the effect of physical activity on circulating cytokines.

Outcome	Method	β (95% CI)	SE	*p*-Value	*p*-FDR
CCL19	Inverse-variance weighted	−0.065 (−0.105, −0.026)	0.020	0.001	0.038
2B4	Inverse-variance weighted	−0.053 (−0.103, −0.003)	0.025	0.037	0.259
CD5	Inverse-variance weighted	−0.060 (−0.109, −0.011)	0.025	0.017	0.175
CD6	Inverse-variance weighted	−0.050 (−0.094, −0.006)	0.022	0.025	0.208
Cystatin D	Inverse-variance weighted	0.045 (0.006, 0.083)	0.020	0.024	0.208
Fractalkine	Inverse-variance weighted	−0.046 (−0.089, −0.003)	0.022	0.037	0.259
IL10RB	Inverse-variance weighted	−0.066 (−0.104, −0.027)	0.020	0.001	0.037
IL15RA	Inverse-variance weighted	−0.056 (−0.099, −0.013)	0.022	0.010	0.127
IL18R1	Inverse-variance weighted	−0.039 (−0.079, 0.000)	0.020	0.049	0.295
MIP-1α	Inverse-variance weighted	−0.051 (−0.090, −0.012)	0.020	0.011	0.127
MMP-10	Inverse-variance weighted	−0.054 (−0.093, −0.014)	0.020	0.008	0.127
PD-L1	Inverse-variance weighted	−0.050 (−0.089, −0.011)	0.020	0.011	0.127
TNFRSF9	Inverse-variance weighted	−0.057 (−0.101, −0.013)	0.022	0.011	0.127

## Data Availability

All data utilized in this study are publicly accessible. Summary statistics for physical activity are available from the GWAS conducted by Klimentidis et al. [14] (DOI:10.1038/s41366-018-0120-3) and can be accessed at https://sites.arizona.edu/arizonageneticepidemiology/data/ (accessed on 3 September 2024). Data for thymic tumors were sourced from the FinnGen consortium (Release 11) and are available at https://finngen.gitbook.io/documentation/data-download (accessed on 3 September 2024). Summary statistics for circulating cytokines can be found in the GWAS by Zhao et al. [36] (DOI:10.1038/s41590-023-01588-w), accessible at https://www.ebi.ac.uk/gwas/publications/37563310 (accessed on 3 September 2024).

## Supplementary Materials

The following supporting information can be downloaded at: https://www.mdpi.com/article/10.3390/ijms252413485/s1.
