# Supplementary material for: Causal Relationship Between Physical Activity and Thymic Tumors Mediated by Circulating Cytokines: A Mendelian Randomization Mediation Analysis
Source: Int J Mol Sci. 2024 Dec 16;25(24):13485. doi: 10.3390/ijms252413485 (PMC11676807; doi:10.3390/ijms252413485)
Supplement: Supplementary file 1 [file ijms-25-13485-s001.zip › Supplementary_Figures.pdf]

Supplementary Figures

Figure S1. Scatter plots of significant estimates from genetically predicted physical activity on (a) benign thymic neoplasms; (b) malignant thymic neoplasms.

Figure S2. Funnel plots of significant estimates from genetically predicted physical activity on (a) benign thymic neoplasms; (b) malignant thymic neoplasms.

Figure S3. Leave-one-out plots of significant estimates from genetically predicted physical activity on (a) benign thymic neoplasms; (b) malignant thymic neoplasms.

Figure S4. Scatter plots of significant estimates from genetically predicted physical activity on (a) IL10RB levels; (b) CCL19 levels.

Figure S5. Funnel plots of significant estimates from genetically predicted physical activity on (a) IL10RB levels; (b) CCL19 levels.

Figure S6. Leave-one-out plots of significant estimates from genetically predicted physical activity on (a) IL10RB levels; (b) CCL19 levels.

Figure S7. Scatter plots of significant estimates from genetically predicted IL10RB levels on benign thymic neoplasms.

Figure S8. Funnel plots of significant estimates from genetically predicted IL10RB levels on benign thymic neoplasms.

Figure S9. Leave-one-out plots of significant estimates from genetically predicted IL10RB levels on benign thymic neoplasms.

a.

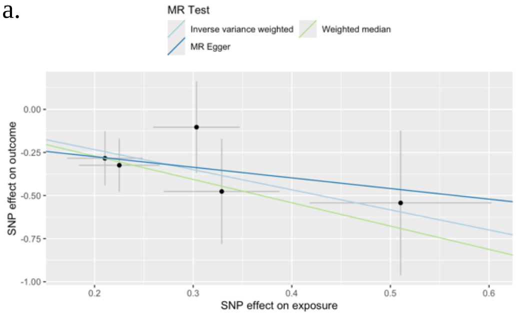

b.

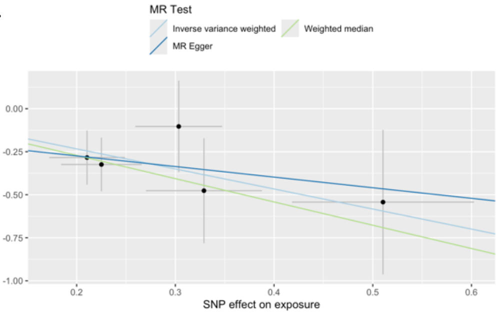

Figure S1. Scatter plots of significant estimates from genetically predicted physical activity on (a) benign thymic neoplasms; (b) malignant thymic neoplasms.

a.

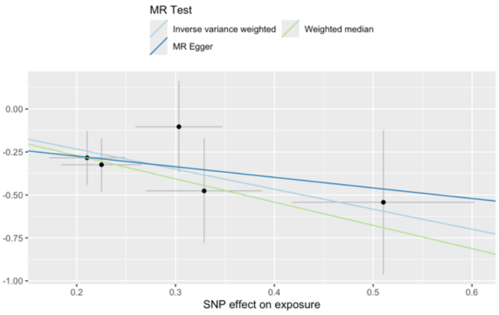

b.

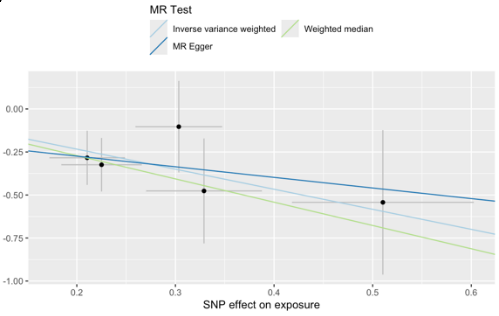

Figure S2. Funnel plots of significant estimates from genetically predicted physical activity on (a) benign thymic neoplasms; (b) malignant thymic neoplasms.

a.

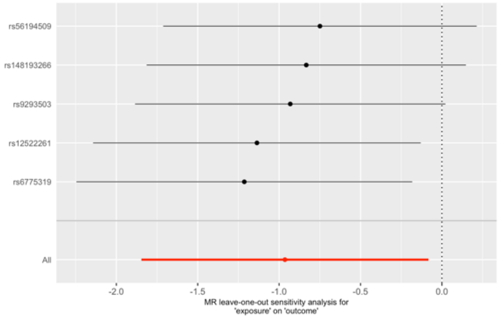

b.

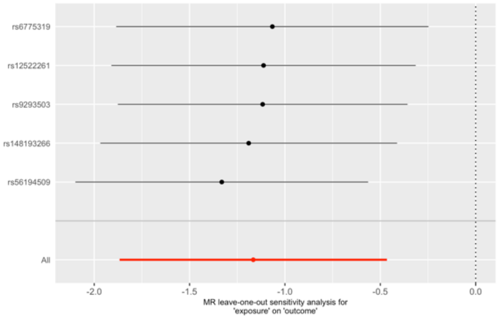

Figure S3. Leave-one-out plots of significant estimates from genetically predicted physical activity on (a) benign thymic neoplasms; (b) malignant thymic neoplasms.

a.

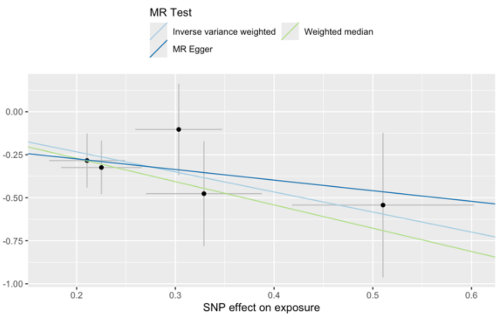

b.

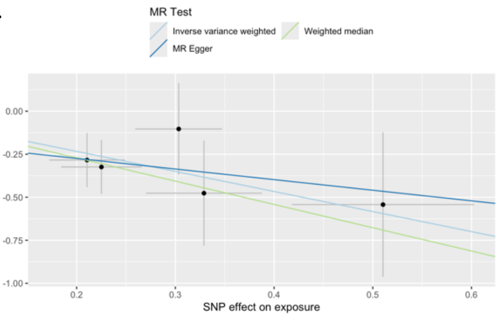

Figure S4. Scatter plots of significant estimates from genetically predicted physical activity on (a) IL10RB levels; (b) CCL19 levels.

a.

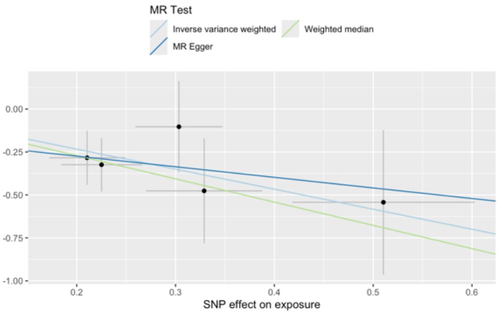

b.

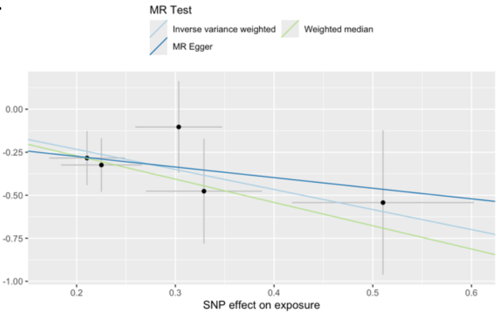

Figure S5. Funnel plots of significant estimates from genetically predicted physical activity on (a) IL10RB levels; (b) CCL19 levels.

a.

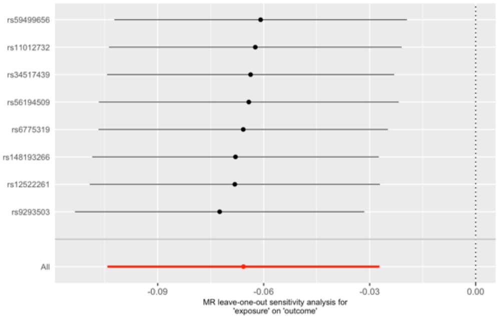

b.

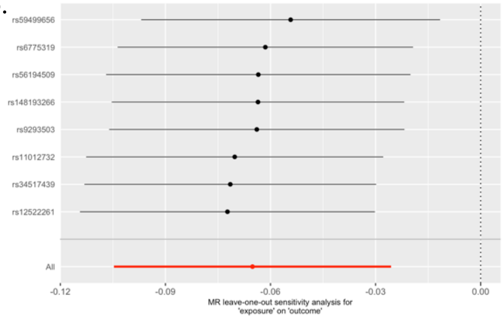

Figure S6. Leave-one-out plots of significant estimates from genetically predicted physical activity on (a) IL10RB levels; (b) CCL19 levels.

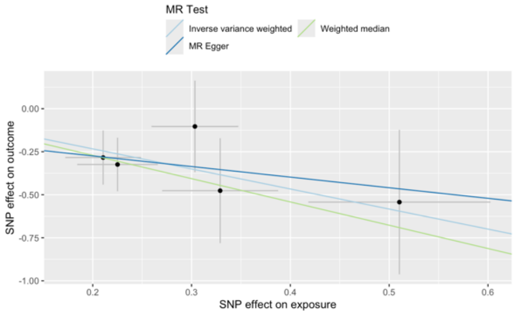

Figure S7. Scatter plots of significant estimates from genetically predicted IL10RB levels on benign thymic neoplasms.

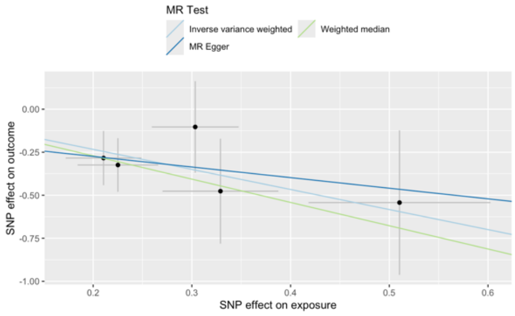

Figure S8. Funnel plots of significant estimates from genetically predicted IL10RB levels on benign thymic neoplasms.

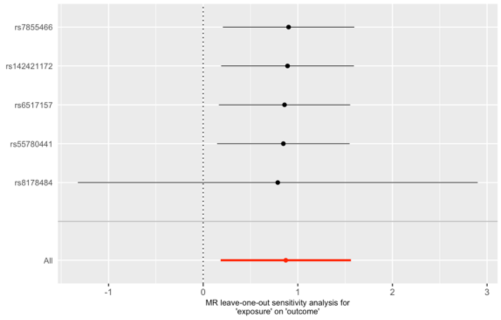

Figure S9. Leave-one-out plots of significant estimates from genetically predicted IL10RB levels on benign thymic neoplasms.
